# Fingerprinting of Peach During the Ripening Process Using an Analytical Platform with Spectrometric and Volatilome-Based Chromatographic Techniques

**DOI:** 10.1021/acs.jafc.5c01524

**Published:** 2025-06-23

**Authors:** Claudia Giménez-Campillo, Natalia Arroyo-Manzanares, Marta Pastor-Belda, Natalia Campillo, Lukas Bodenbender, Philipp Weller, Pilar Viñas

**Affiliations:** † Department of Analytical Chemistry, Faculty of Chemistry, 16751 University of Murcia, Regional Campus of International Excellence “Campus Mare Nostrum”, E-30100 Murcia, Spain; ‡ Faculty of Biotechnology, Institute for Instrumental Analytics and Bioanalytics, 120168 Technische Hochschule Mannheim, 68163 Mannheim, Germany

**Keywords:** peach, ripening, volatilome, FT-NIR, HS-GC–MS, HS-GC–IMS

## Abstract

In recent years, the quality of peaches has been related to their early harvest, so this work has focused on the characterization of the spectral fingerprint using Fourier transform near-infrared spectroscopy, and headspace gas chromatography (HS-GC) coupled to ion mobility spectrometry (IMS) and mass spectrometry (MS) based on their volatilome or volatile organic compound content, with the aim of identifying the optimum ripening point of peaches. A total of 344 samples of two different varieties at all ripening stages were analyzed. The principal component analysis (PCA) showed a clear tendency for samples at the same stage of ripening to form visible clusters. The groups identified by PCA were used to construct partial least-squares discriminant analysis models that allowed differentiation according to ripening and variety. The overall results were very promising, especially for the volatilomes measured by HS-GC–IMS and HS-GC–MS.

## Graphical abstract

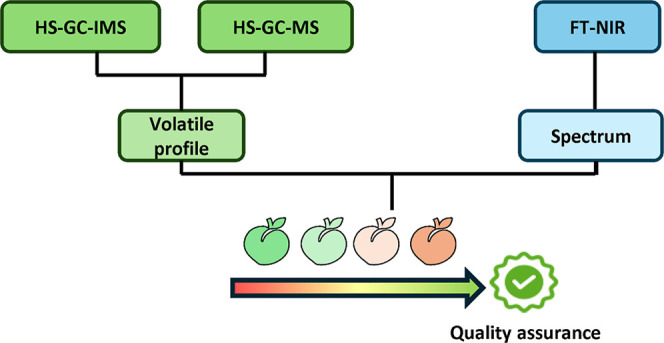

## 1 Introduction

Peach, along with nectarine, plum, apricot, cherry, and almond, belongs to the genus (genus described by Charles Linnaeus in 1753).^1^ Originally from China, it is the second most important fruit in Europe after apple,^2^ is of great economic importance, and is consumed fresh, canned and in juice. It is rich in vitamins, minerals, and antioxidants. Currently, in addition to China, there are other world leaders in the production of this fruit, including Spain, Italy, Turkey, and Greece.^3^

In recent decades, consumer complaints about poor fruit quality have often been the result of premature harvesting for commercial reasons. This practice, while facilitating transport and distribution by extending the fruit’s presence in the market, has a negative impact on its flavor, texture, and nutritional value. Consequently, ripening monitoring and control have become an important part of fruit management.^4^ Despite extensive research on the physicochemical properties of peaches during the ripening stage to obtain adequate maturity indices, objective indicators of consumer acceptance are still lacking.^5^ Ripening time has been shown to be directly related to flavor characteristics, which are influenced by sugar accumulation, acidity, and the production of low-molecular-weight volatile organic compounds (VOCs),^6^ also called volatilomes. Therefore, VOCs can be a key indicator in peach quality assessment. Understanding exactly which VOCs are responsible for aroma at different ripening stages can help to develop rational and objective methods to ensure high-quality fruit, both in consumption and processing.^7^ This approach would not only be beneficial for consumers, who would receive products with optimal flavor, texture, and nutritional value, but also would support producers.

A literature review reveals the existence of several studies on the characteristic volatile profile of peaches.^1,8–14^ However, it is important to note that there is a significant lack of research on the variation of these compounds during ripening.^15^ Most of the works on VOC determination used gas chromatography coupled to mass spectrometry (GC–MS)^1,6,8–19^ and solid-phase microextraction as the sample preparation technique.^6,9–12,15–17,19^ In recent years, ion mobility spectrometry (IMS) has been used as an alternative technique for the determination of VOCs in peach.^11,12,18,20,21^ The aim of these studies was to investigate the volatile profile,^11^ the differentiation between varieties,^18^ and the influence of packaging and storage of this fruit.^20,21^ Therefore, the current state-of-the art techniques reveal that GC–MS and GC–IMS techniques have not been applied to predict peach ripening. It has been shown that it is possible to predict optimal ripening by near-infrared spectrometry (NIR) in relation to the measurement of different parameters, such as soluble solid content,^22–28^ flesh firmness,^23,26,28,29^ pectin content,^30^ dry matter,^23–25^ absorbance difference index,^25,26^ and acidity.^27^

The objective of this work was the development of methodologies for the prediction of the ripening stage of peaches and, therefore, of their quality for the consumer by means of a simple analysis based on their VOC content, applying headspace GC (HS-GC)–MS and HS-GC–IMS techniques and on their Fourier transform near-infrared spectroscopy (FT-NIR) spectral fingerprint. The novelty of the study lies in the fact that correlations have not been established with other chemical parameters measured in peaches, but rather chemometric models based on an analytical platform have been applied to obtain relevant information on the ripening of this fruit. The studies were conducted on two different peach varieties (yellow-fleshed and red-fleshed) grown in one geographical origin of the agricultural sector (Jumilla, Region of Murcia, Spain), which have not been previously analyzed, and chemometric models were also applied to discriminate between these two varieties.

## 2 Materials and Methods

### 2.1 Samples and Reagents

Two different peach varieties from Jumilla (Region of Murcia, Spain), yellow-fleshed and red-fleshed, were sampled weekly throughout their ripening process from June to October in the 2023 season. The two varieties have been studied to increase diversity, as they have a difference of 2–3 weeks in reaching the optimum ripeness and therefore harvesting of each variety. The yellow-fleshed variety was sampled from mid-June to mid-September, while the red-fleshed variety was sampled from mid-June to early October in order to obtain peaches at all stages of ripeness. Samples were generously provided by local growers.

A total of 344 peaches were analyzed, of which 154 belonged to the yellow-fleshed variety and 190 to the red-fleshed variety. Peaches (5–6 pieces) were collected weekly in the same field in Jumilla from different trees to obtain a representative sample on each date. To ensure uniform conditions for analysis, all peaches were frozen at −20 °C immediately after sampling and up to 8 h before the start of analysis, thawed in batches to ensure equal thawing time. The peaches were then completely homogenized in a Bosch blender (Stuttgart, Germany) immediately prior to analysis to avoid oxidation. During the crushing process, no distinction was made between the pulp and the skin, thus obtaining a homogeneous puree that would facilitate subsequent evaluation of the samples in a single analysis. This procedure was adopted to obtain a representative and complete profile of all the VOCs present in the whole peaches,^2,31^ since previous studies have shown that VOC composition varies between the skin and pulp.^15^

Several standards supplied by Fisher Scientific (Loughborough, UK) and Sigma-Aldrich (St. Louis, MO, USA) were used for the identification and quantification of volatile compounds, including alkanes (decane, tetradecane), alcohols (2-phenylethanol, isopentyl alcohol, hexan-1-ol, (*Z*)-hex-3-en-1-ol, (*Z*)-hex-2-en-1-ol, heptan-2-ol, octan-1-ol, nonan-1-ol), aldehydes (pentanal, (*E*)-pent-2-enal, furfural, hexanal, (2*E*,4*E*)-hepta-2,4-dienal, octanal, (*E*)-oct-2-enal, nonanal, (*E*)-non-2-enal, decanal, undecanal, benzaldehyde), ketones (heptan-2-one, β-ionone, and 6-methylhept-5-en-2-one), esters (ethyl acetate, ethyl hexanoate, hexyl acetate, and (*Z*)-hex-3-enyl acetate), terpenes (limonene, *p*-cymene, linalool, terpinolene, and γ-terpinene), and furans (2-pentylfuran). Finally, a mixture of alkanes ranging from C8 to C40 was used to calculate the Kovats index of each identified peak, and a mixture of ketones ranging from C4 to C9 was used to determine the retention index (RI) in the GC–IMS identification. This mixture of alkanes at a concentration of 500 μg mL^–1^ together with pure ketone standards (butan-2-one, pentan-2-one, hexan-2-one, heptan-2-one, octan-2-one, and nonan-2-one) and sodium chloride was supplied by Sigma-Aldrich.

### 2.2 Instruments, Analytical Procedures, and Data Processing

#### 2.2.1 FT-NIR Analysis

FT-NIR analysis was performed using a FT-NIR multipurpose analyzer spectrophotometer, equipped with a solid sample compartment, from Bruker Optik GmbH (Ettlingen, Germany).

A weight of 0.5 g of homogenized peach was placed in a vial and measured by FT-NIR in the reflectance mode. Before each analysis, a background scan was performed, and the measurement was repeated five times. Spectral data covered the wavenumber range from 12,500 to 3600 cm^–1^ with a resolution of 8 cm^–1^ and are expressed in units of absorbance. Each spectrum was obtained from 32 scans, and the average of the five measurements for each peach sample was calculated to obtain a representative spectrum.

The FT-NIR software used to process the data was OPUS software, version 8.5. In addition, the data set obtained was subjected to three transformations using Unscrambler X software. Multiplicative scattering correction was applied to correct for the effects of particle-size-induced light scattering effects. Next, Savitzky–Golay smoothing was applied (window = 9, polyorder 2, first derivative) to reduce the inherent instrumental noise and to correct for the additive and multiplicative effects present in the spectrum. The full spectrum (2224 features) was used as a variable in the chemometric models constructed from the FT-NIR data. This approach was adopted in order to make use of all available spectral information given the observed variations between the samples across the entire range studied.

#### 2.2.2 HS-GC–IMS Analysis

For GC-IMS analysis, a 6890N gas chromatograph (version N.05.05) from Agilent Technologies (Waldbronn, Germany) was coupled to a G.A.S. (Gesellschaft für Analytische Sensorsysteme) IMS module (Dortmund, Germany). A multipurpose sampler (MPS) from Gerstel GmbH (Mühlheim, Germany) was used, configured for headspace mode and injecting the sample in the gas phase, using a 2.5 mL syringe. The carrier gas was nitrogen with a purity of 99.99%, operated at a constant flow rate of 1 mL min^–1^, and supplied by Air Liquide (Madrid, Spain). An Agilent HP-5MS UI capillary column (0.25 mm × 30 m with a film thickness of 0.25 μm), consisting of 5% diphenyl and 95% dimethyl polysiloxane, was used for the analysis.

A glass vial with a magnetic stopper containing 2 g of homogenized sample was placed in the MPS. Sample treatment consisted of incubating the sample at 80 °C for 5 min at a stirring speed of 750 rpm. After this time, 750 μL of the headspace volume was injected with the 2.5 mL syringe into the GC inlet port heated at 80 °C in splitless mode. The oven program started with an initial temperature of 50 °C for 4 min and then increased at a rate of 10 °C min^–1^ to 130 °C, which was maintained for 8 min, resulting in a total run time of 20 min. The transfer line to the IMS cell was maintained at 110 °C, which was operated with nitrogen as buffer gas at a flow rate of 150 mL min^–1^ and featured an atmospheric ^3^H-based ion source (<300 MBq) operating in positive-ion mode. The IMS module had a 98 mm drift tube operated at 80 °C and used a constant voltage of 500 V cm^–1^. Each spectrum was averaged from 32 scans, and the repetition rate was 30 ms. The drift and block voltages were 241 and 50 V, respectively, and the grating pulse duration was 150 μs.

Data generated by HS-GC–IMS were imported, preprocessed, and analyzed using the GC–IMS-tools toolbox based on Python 3.8.8, as described by Christmann et al.^32^ Detailed steps of preprocessing were as follows: a binning with factor 2 was applied to the raw spectra, the drift time axis was aligned to the reactant ion peak (RIP), and the data points were set relative to the RIP signal.^33^ Afterward, the data range with relevant sample information was cropped. This resulted in a drift time axis of 1.025 and 2.2 ms and in a retention time axis of 175–1200 s. Subsequently, asymmetric least-squares background correction, Pareto scaling, and mean centering were applied to the data set. All GC–IMS data were used in an untargeted manner without feature selection.

#### 2.2.3 HS-GC–MS Analysis

GC–MS analysis was carried out on an Agilent Technologies (Santa Clara, CA, USA) model 8890 gas chromatograph and an Agilent 5977B quadrupole mass spectrometer equipped with an inert ion source. A Gerstel MPS operating in headspace mode was used for sample injection.

A mixture containing 2 g of the homogenized sample and 0.75 g of NaCl was prepared in a glass vial with a magnetic stopper and placed in the MPS. The incubation program was 15 min at a temperature of 120 °C with a stirring speed of 750 rpm. A gas volume of 1500 μL of the headspace was injected into a GC inlet port programmed at 140 °C in a 1:10 split mode. The analytes were separated with a continuous helium flow of 1 mL min^–1^ on an identical column as used for the GC–IMS analysis.

The GC oven temperature was programmed as follows: the initial temperature was set at 40 °C (held for 5 min) and then increased to 130 °C at a rate of 5 °C min^–1^, finally reaching 250 °C at a rate of 35 °C min^–1^, for a total run time of 26.5 min. Temperatures of 230, 250, and 150 °C were set for the ion source, transfer line, and quadrupole, respectively. The mass spectrometer, equipped with an electron ionization (EI) source, was operated at a voltage of 70 eV in the full-scan mode over the mass range of 20–400 *m*/*z*.

GC–MS data processing was performed by using MS-DIAL version 4.80 for Windows. The minimum amplitude value was set at 1000, and a total of 427 features were detected among all peach samples. The smoothing level was set to 3, the average peak width to 20, and the sigma window value to 0.5. For identification, a mixture of alkanes (C8 to C40) was injected at a concentration of 5 μg mL^–1^ under the same conditions as the peach samples to calculate the RI of each peak using the Kovats method. For identification, a RI tolerance of 10, a retention time tolerance of 0.5 min, a mass difference (*m*/*z*) of 0.5 Da, and a minimum match threshold of 70% were set. Finally, for alignment, a retention time tolerance of 0.075 min and a minimum similarity of 70% were set.

### 2.3 Chemometric Tools

A number of chemometric tools were used to discriminate between peach varieties and to study the evolution of the ripening stages of the peaches. With these objectives, the preprocessed data obtained from the application of the three methods were treated by principal component analysis (PCA) and partial least-squares discriminant analysis (PLS-DA) using Python 3.9.

Prior to the construction of chemometric models, feature scaling was applied using the StandardScaler from the scikit-learn library to ensure that all features were of equal importance in the model construction. In addition, the residual N-plot was used to check that the data followed a normal distribution in all cases.

The PCA chemometric models were developed by using the 344 samples in a single set. The PLS-DA models were built by dividing the total number of samples into two subsets based on the Kennard–Stone approach: 80% (275 samples) were used for training and the remaining 20% (69 samples) for validation. Further, additional PLS-DA models were developed specifically for the yellow-fleshed variety samples (154 samples) or for the red-fleshed variety (190 samples). In these cases, the splitting rate was also 80% and 20%, with 123 training and 31 validation samples for the yellow-fleshed variety and 152 training and 38 validation samples for the red-fleshed variety. The number of PLS components was evaluated by the root-mean-square error (RMSE) value, dependent on the number of components to reach a local minimum. All subsets were stratified to ensure a comparable class distribution in the test and training sets.

In order to evaluate the results obtained from the PLS-DA models, the following parameters were analyzed for: accuracy, precision, specificity, and sensitivity.

The accuracy of a model is defined as the proportion of correct predictions and is calculated using eq 1, where TP is the number of true positives, TN is the number of true negatives, and *N* is the total number of samples.

1 $$Accuracy=\frac{TP+TN}{N}$$

The precision of the model is defined as the proportion of correct positive predictions and is calculated by using eq 2, where FP is the number of false positives.

2 $$Precision=\frac{TP}{TP+FP}$$

Finally, specificity and sensitivity were calculated on an individual basis for each class (*k*) of the data set, as the performance of the model often varies from class to class. The specificity of the model is defined as the proportion of correctly identified negatives and is calculated using eq 3.

3 $$Specificity=\frac{{TN}_{k}}{{TN}_{k}+{FP}_{k}}$$

The sensitivity of the model is defined as the proportion of correctly identified positives and is calculated using eq 4, where FN is the number of false negatives.

4 $$Sensitivity=\frac{{TP}_{k}}{{TP}_{k}+{FN}_{k}}$$

For validation of all models and to evaluate the model performance, cross-validation (CV) was performed using 5 different validation sets (*k* = 5), and the respective figures of merit (RMSEC and RMSECV) were generated. Specifically, CV was carried out by dividing the training set into five equal-sized subsets (folds). In each of the five iterations, four of the folds were used to train the model, while the remaining fold was used to validate it. By the end of the process, each sample in the training set will have been used once as the validation set. Random shuffling and a fixed seed were implemented to ensure the reproducibility of this technique. Average performance metrics and their standard deviation (SD) are obtained from these iterations, enabling the stability of the model to be assessed and overfitting to be prevented. Finally, the model trained using the entire training set was evaluated using an external validation set comprising 20% of the data, which had not been used during model building.

## 3 Results and Discussion

### 3.1 Optimization of the Analytical Procedures

The optimization required to apply the FT-NIR technique was very simple. The resolution was optimized by performing experiments at 4, 8, and 16 cm^–1^. Since higher resolutions increased the analysis time without significantly improving the spectra, an optimal resolution of 8 cm^–1^ was chosen. The optimal number of scans was then investigated by testing 32 and 64 scans. No significant improvement was observed as the number of scans increased, confirming that 32 scans was the optimal number for fast analysis.

The analytical procedure for applying the HS-GC–IMS methodology was optimized to obtain the best results in peak separation and intensity. The optimization included the evaluation of the sample amount, salt presence, oven program, and incubation time and temperature.^34^ The sample amount was investigated in the range of 0.5–3 g, with the highest values for the signal intensity and number of peaks found using 2 g of sample, which was selected (Figure S1A). The possible effect of salt incorporation on the increase of VOC signals was also investigated, covering a range from 0 to 1 g of NaCl. The data showed a decrease in some signals when the sample contained a certain percentage of salt, so the addition of salt was discarded. A study of the oven program was carried out to optimize the VOC separation in the shortest time. Following the recommendations of the IMS manufacturer, temperatures above 130 °C were avoided in the oven program in order to prevent condensation of the VOCs, as the drift tube had a limited temperature of 100 °C. Therefore, the oven temperature program was set to start at 50 °C and increase the temperature by 10 °C min^–1^ until 130 °C was reached and maintained for 8 min. A slight accumulation of compounds was observed at the beginning of the topographic map, so it was decided to maintain the initial temperature of 50 °C for 4 min to improve the separation. The effect of sample incubation temperature was investigated in the range of 60–90 °C; higher temperatures were not tested due to manufacturer restrictions on this parameter. An increase in the number of signals was observed up to 80 °C; therefore, this temperature was selected (Figure S1B). The sample incubation time was studied in the range from 2 to 45 min, and it was found that 5 min was sufficient to obtain better signals. The temperature of the drift tube was also investigated in the range of 60–90 °C, and an increase in both the intensity and number of VOC signals was observed at 80 °C (Figure S1C).

Peach analysis by HS-GC–MS was previously developed by our research group;^31^ therefore, all the parameters were adopted.

Before collecting and analyzing the samples, a study was conducted to evaluate the stability of the volatile profile after freezing and thawing. The results showed a slight decrease in the VOC intensity without any significant loss of compounds or relevant alterations to the qualitative composition of the aromatic profile. This effect was considered acceptable, since the comparison between samples was not affected by it, and the rapid evolution of the volatile profile of peach, a highly perishable fruit, was slowed down, thus avoiding variations attributable to storage or ripening time. Freezing the samples enabled simultaneous analysis under consistent instrumental conditions, mitigating variability caused by changes in the equipment over time. This strategy had previously been described in the literature for analyzing peach samples.^1,4,11^

### 3.2 Spectral Peach Fingerprint Using FT-NIR

The spectral fingerprints of the peaches analyzed showed very similar profiles, with differences in the absorbance values. Figure 1 shows the FT-NIR spectra of the average raw data obtained from all peach samples collected at different stages of ripeness, recorded in the range 12,500–3600 cm^–1^. It is observed that as the ripening stage progresses, the absorbance increases in the range 11,800–3600 cm^–1^.

**Figure 1:**
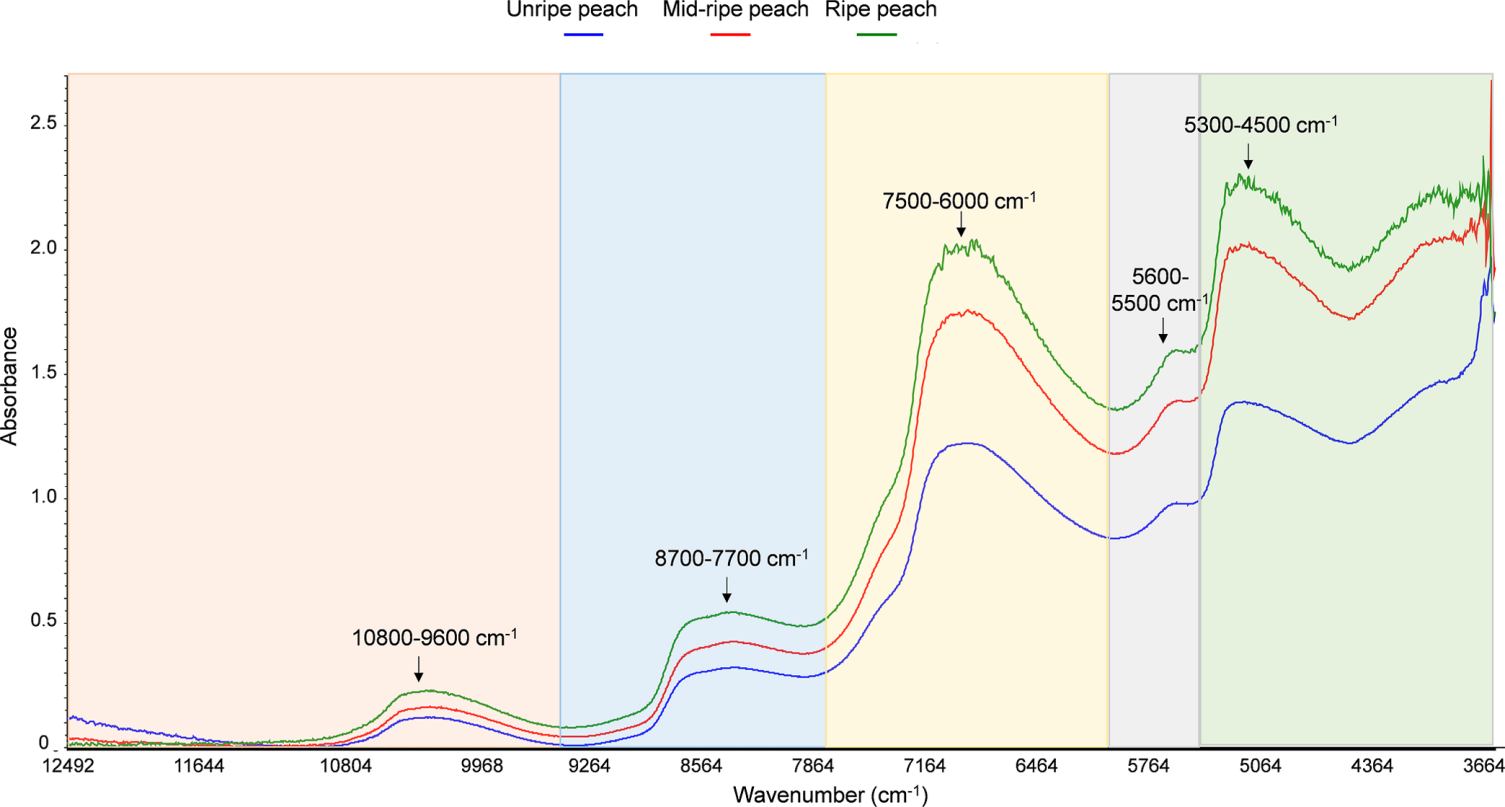
Raw FT-NIR spectra of the average of all peach samples at different stages of ripening. The figure shows the FT-NIR spectra corresponding to the average of peach samples at different stages of ripeness: the blue spectrum represents unripe peaches, the red spectrum also represents unripe peaches but in a more advance ripeness process, and the green spectrum represents ripe peaches ready for consumption. Also, the most representative absorption bands of the peach observed in the spectra are indicated.

In the spectrum of the different peach samples, four different regions can be identified according to the NIR bands: 12,500–9400 cm^–1^, 9400–7700 cm^–1^, 7700–5800 cm^–1^, and 5800–3600 cm^–1^. Peach samples contain mainly water (85–95%) in their composition, and therefore characteristic water bands were found from right to left, including bands with different absorbances. In the first region, from 5800 to 3600 cm^–1^, there is a broad absorption band with a maximum at around 5160 cm^–1^, which corresponds to the first combination of asymmetric stretching and bending of the water molecule. Also, in this region, there is another narrow and weaker combination band at 5620 cm^–1^, associated with the bending, asymmetric stretching, and intramolecular modes of the water molecule. In the region between 7700 and 5800 cm^–1^, a broad band is observed at 6880 cm^–1^ involving the symmetric and asymmetric stretching modes of the water molecule, known as the first overtone of OH stretching. Between 9400 and 7700 cm^–1^, there is a second weak combination band involving the symmetric stretching, asymmetric stretching, and bending modes of the water molecule. Finally, in the region from 12,500 to 9400 cm^–1^, an absorption band related to the asymmetric and symmetric stretching modes of the water molecule is observed, called the second overtone of OH stretching.^35^ The spectra show that the maximum absorbance value of each band increases as the ripening stage progresses. This correlation suggests that the ripening process in peaches is associated with an increase in water content, which affects the texture, weight, and possibly the flavor of the fruit.

In addition, it is important to mention that some of the bands described could be related to the presence of C–H bonds in compounds such as alkanes, sugars, and aromatics, although in smaller amounts compared to water. The band at 5620 cm^–1^ could be related to the first overtone of C–H stretching associated with methyl and methylene groups, which are characteristic of carbohydrates like glucose and fructose. The concentrations of these compounds increase during maturation. Conversely, the band at 8310 cm^–1^ could correspond to the second overtone of C–H stretching,^35^ which is associated with the increasing presence of sugars. Finally, an additional signal around 10,210 cm^–1^ could be attributed to the third overtone of the C–H bond, although its relative intensity is low. These C–H bond-related bands complement the water signals, allowing a direct correlation to be established between the NIR spectrum and key ripening parameters such as soluble sugar content and acidity. Therefore, FT-NIR spectral analysis reflects not only the evolution of water content but also changes in the fruit’s chemical composition, offering a nondestructive tool for assessing the ripening stage of peaches, as described in numerous studies.^23,25,26^ A summary of all of the bands identified in peach samples at different maturity levels is presented in Table 1.

**Table 1:** Absorption Bands Presented in the Peach Samples^a^

region (cm^–1^)	maximum (cm^–1^)	link (cm^–1^)	assignment	name
12,500–9400	10,210	O–H	2 ν_1_ + ν_3_	second overtone O–H
	10,210	C–H		third overtone C–H
9400–7700	8310	C–H		second overtone C–H
	8310	O–H	ν_1_ + ν_2_ + ν_3_	second combination O–H
7700–5800	6880	O–H	ν_1_ + ν_3_	first overtone O–H
5800–3600	5620	C–H		first overtone C–H
	5620	O–H	ν_2_ + ν_3_ + ν_L_	first combination O–H
	5160	O–H	ν_2_ + ν_3_	first combination O–H
	5160	C–H		combination band C–H

a ν_1:_ symmetric stretch of the water molecule; ν_2_: bending mode of the water molecule; ν_3_: asymmetric stretch of the water molecule; ν_L_: intermolecular mode of the water molecule.

### 3.3 Identification and Quantification of Volatile Compounds Using GC–MS and GC–IMS

The volatile fingerprint of the peach samples was established by using GC coupled to MS and IMS detectors. Figures 2 and 3 show a topographical map using IMS and a chromatogram obtained by the MS detector for a peach sample, respectively. In both cases, identification of the VOCs was carried out.

**Figure 2:**
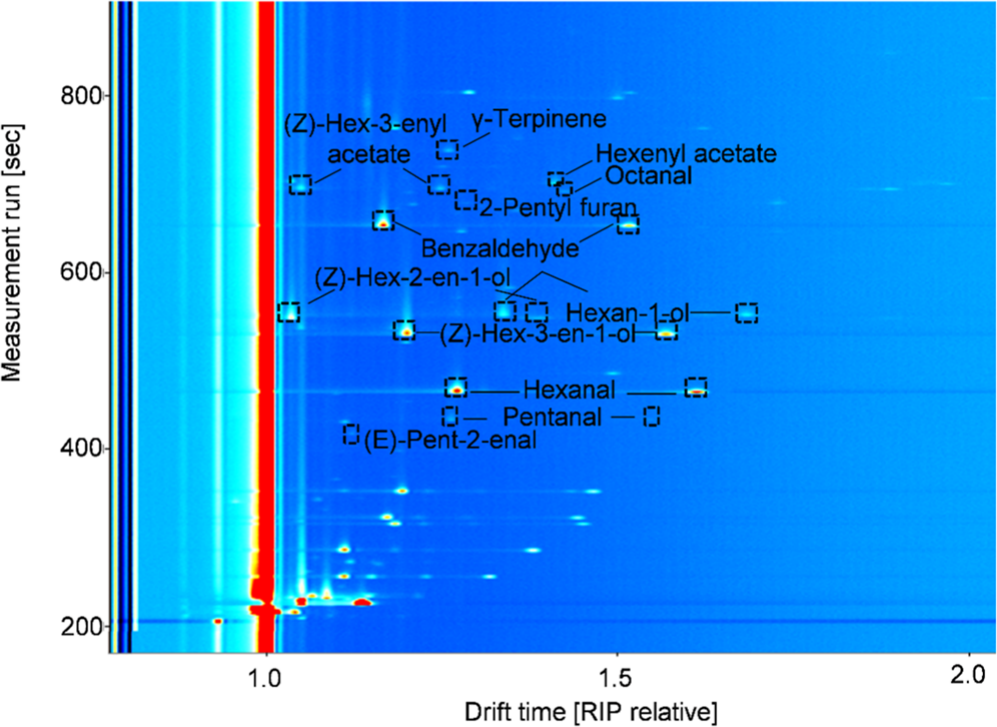
Topographic map of a peach sample. The figure shows a topographical map obtained by HS-GC–IMS, indicating the VOCs identified and verified in peach samples.

**Figure 3:**
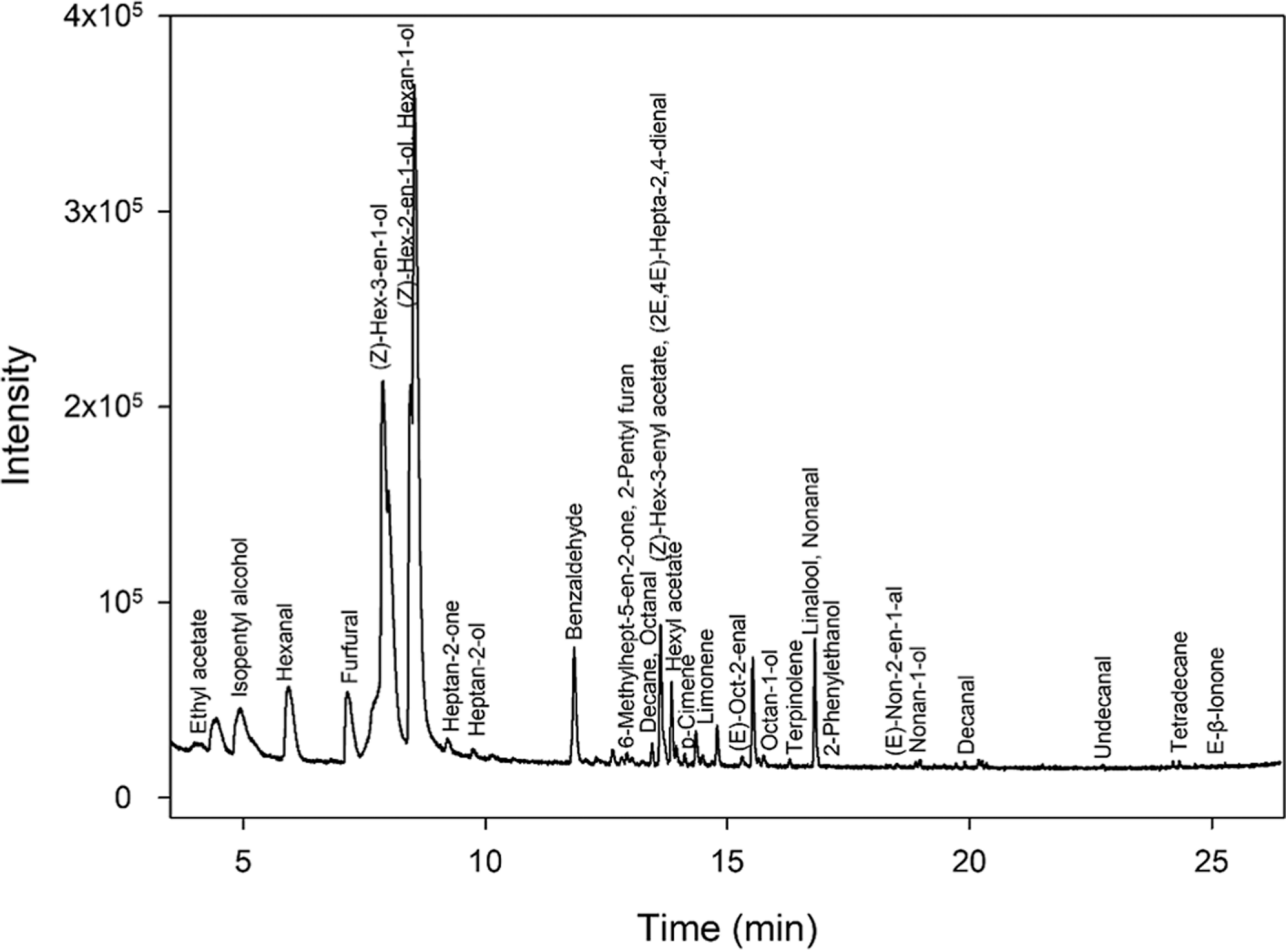
Chromatogram of a peach sample. The figure shows a chromatogram obtained by HS-GC–MS, indicating the compounds identified and verified in peach samples.

Untargeted studies were performed by processing the raw data from the HS-GC–MS analysis of the samples using MS-DIAL software under the conditions described in Section 2.2.3. A total of 427 features were extracted from the peach samples, taking into account the different varieties and the whole ripening process.

HS-GC–IMS was used to obtain the topographical maps of all samples. The GC–MS metabolomic MSP spectral library and the GAS IMS library allowed putative identification of some of the features detected.

Some of the putatively identified compounds were confirmed by both techniques using commercial analytical standards (described in Section 2.1) prepared in water at a concentration of 5 μg mL^–1^. Table 2 shows the compounds identified and confirmed using HS-GC–MS and HS-GC–IMS. Some volatile compounds were detected by both techniques, while others were detected by only one technique. For instance, γ-terpinene was identified only by HS-GC–IMS, and 2-phenylethanol was detected only by HS-GC–MS. These discrepancies can be explained by the inherent differences between the two techniques, including their separation mechanisms (ionic mobility versus mass-to-charge ratio), their chromatographic conditions, their retention times, and their detection and quantification thresholds. These characteristics make HS-GC–IMS more sensitive to certain light volatile compounds, while HS-GC–MS offers greater capability to identify the structure of less volatile compounds. Therefore, using both techniques together allowed us to obtain a broader, more representative volatile profile of peaches, reinforcing the complementarity of the analytical approach used in this study.^36,37^

**Table 2:** Compounds Identified in the Samples by GC–IMS and GC–MS

	HS-GC–IMS			HS-GC–MS	
compound	*R*_T_ (s)	*D*_Tmonomer_ (ms)	*D*_Tdimer_ (ms)	*R*_T_ (min)	main *m*/*z*
ethyl acetate				4.190	43
isopentyl alcohol	396.00	8.972	11.105	4.907	55
(*E*)-pent-2-enal	416.79	8.066	10.119		
pentanal	431.64	9.060	11.149		
hexanal	460.35	9.142	11.662	5.930	44
furfural				7.142	96
(*Z*)-hex-3-en-1-ol	529.65	8.488	11.209	8.049	41
(*Z*)-hex-2-en-1-ol	544.50	7.434	9.982	8.485	57
hexan-1-ol	544.50	9.582	12.103	8.570	56
heptan-2-one	574.20	9.077		9.399	58
heptan-2-ol	580.14	12.609		9.750	55
benzaldehyde	642.51	8.368	10.902	11.828	106
6-methylhept-5-en-2-one	666.27	8.688		12.922	43
2-pentyl furan	667.27	9.262		13.027	81
decane				13.317	57
ethyl hexanoate	681.12	9.648		13.354	88
octanal	681.12	10.235		13.444	56
(*Z*)-hex-3-enyl acetate	686.07	7.451	8.995	13.623	67
(2*E*,4*E*)-hepta-2,4-dienal				13.696	81
hexyl acetate	700.92	10.111	14.274	13.852	43
*p*-cymene	704.88	8.968		14.125	119
limonene	706.86	9.008	9.528	14.257	68
γ-terpinene	740.52	8.981			
(*E*)-oct-2-enal				15.303	70
octan-1-ol				15.743	56
terpinolene				16.294	117
linalool	788.04	8.981		16.664	93
nonanal	786.06	10.782	14.557	16.808	57
2-phenylethanol				17.091	91
(*E*)-non-2-en-1-al				18.522	70
nonan-1-ol				18.885	56
decanal				19.900	57
undecanal				22.770	57
tetradecane				24.330	57
*E*-β-ionone				25.167	177

Most of the compounds identified were detected in more than 95% of the samples analyzed, except for *E*-β-ionone (67.7%), (*E*)-non-2-en-1-al (46.2%), γ-terpinene (30.3%), ethyl hexanoate (16.9%), (*E*)-pent-2-enal (15.1%), and pentanal (2.62%). It should be noted that the difference in the presence of each compound between the different varieties analyzed does not usually exceed 10%, except in the case of (*E*)-non-2-en-1-al, which shows a remarkable variation of 35%.

Some of the compounds identified in this study have previously been described in the literature as undergoing significant changes throughout the peach ripening process. In particular, it has been reported that esters, such as hexyl acetate, predominate in the final stages of ripening. Conversely, alcohols and C6 aldehydes, such as hexanal, are more prevalent in the early stages and decrease as the level of ripening progresses. Similarly, the linalool concentration progressively increases throughout the ripening process.^5,15^

### 3.4 Chemometric Model for the Discrimination of Peach Varieties

Ripening studies were carried out by studying two different peach varieties, yellow-fleshed and red-fleshed. Since no previous studies had been carried out on these peach varieties, PLS-DA models were applied to discriminate between these two varieties. For this purpose, the preprocessed data (described in Section 2.2) of spectroscopic profiles by the FT-NIR technique and volatile profiles by HS-GC–MS and HS-GC–IMS were used.

The PLS-DA models were developed using all the features extracted for FT-NIR (full spectra = 2224 features) and for GC–MS (427 features). For GC-IMS data, all data from the preprocessed data according to Section 2.2.2 were used. Figure 4 shows the PLS-DA model score plots for the three analytical techniques, and Table 3 shows the parameters extracted from the models to assess their quality for each instrument.

**Figure 4:**
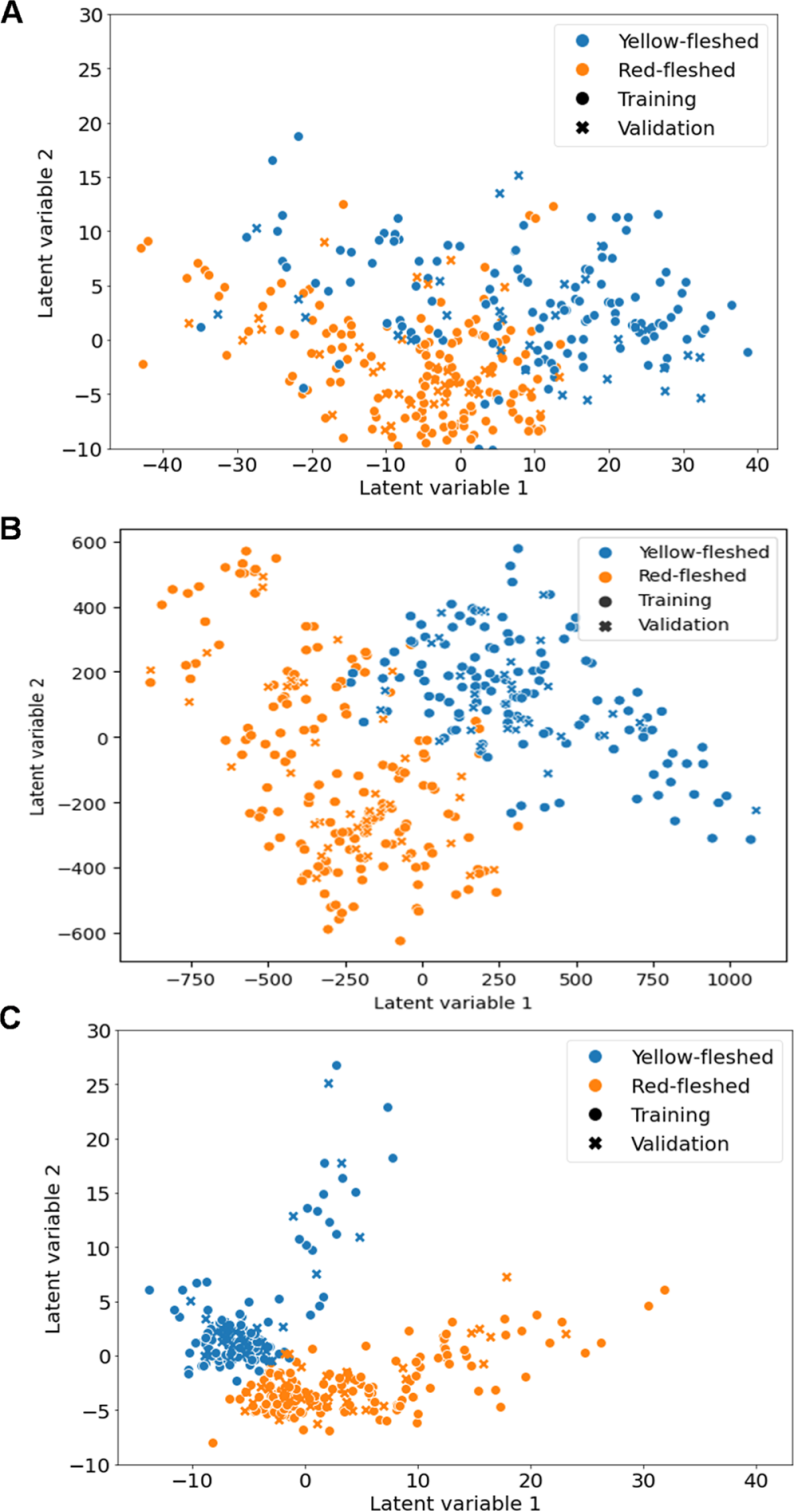
PLS-DA score. Score plot obtained from the PLS-DA model built to differentiate between yellow-fleshed and red-fleshed varieties: (A) FT-NIR, (B) HS-GC–IMS, and (C) HS-GC–MS.

**Table 3:** Quantitative Parameters of PLS-DA Models Built to Differentiate between Peach Varieties^a^

instrument	components	accuracy	precision	specificity	sensitivity	% *T*	CV (mean ± SD)
FT-NIR	5	93.9	93.9	93.7	93.9	95.6	88.4 ± 5.5
HS-GC–IMS	7	98.3	98.3	98.2	98.3	100	98.5 ± 0.9
HS-GC–MS	5	99.4	99.4	99.5	99.4	99.3	98.6 ± 1.4

a % *T*: training rate; CV: cross-validation, SD: standard deviation.

All three PLS-DA models show performance parameter values (accuracy, precision, specificity, and sensitivity) above 90%, indicating good overall performance. However, in the case of the FT-NIR model, a slightly lower CV percentage of 90% is observed, which, although still a very good result (88.4%), indicates that the new samples fit the training model slightly worse than that in the other cases. On the other hand, the models built using VOCs as features (HS-GC–IMS and HS-GC–MS) show very high and balanced training and CV rates (≥98.5), which show the good generalizability of both models, demonstrating their robustness and reliability when applied to new data.

To complete the analysis and identify the variables with the greatest influence on the discrimination between the two peach varieties, the values of importance in projection (VIP) obtained from the PLS-DA models were analyzed. Table S1 shows the five features with the highest VIP value in each model. For each of the most important volatile compounds, the analytical characteristics are presented: retention time and drift time for IMS, retention time together with the main ion (*m*/*z*) for MS in the case of unknown compounds, and their name if they have been previously identified in Section 5.3. It should be noted that both the pentanal monomer and dimer have been identified as markers of great importance in distinguishing between yellow-fleshed and red-fleshed varieties. In order to perform the HS-GC–IMS identification, it is necessary to refer to Figure S2, which is generated by the software and shows the VIP scores of the model.

In conclusion, although all models are very promising, both HS-GC–IMS and HS-GC–MS give slightly better results in the PLS-DA models for peach variety discrimination than the models based on FT-NIR data. This is due to the high sensitivity and specificity of HS-GC–MS and HS-GC–IMS, which allow accurate identification of individual volatile compounds. In contrast, FT-NIR analysis is an analytical technique that produces less specific and detailed data, resulting in lower sample classification performance.^38,39^

### 3.5 Chemometric Model for the Study of the Ripeness Stage of Peaches

To carry out the peach ripening study, PCA models were initially developed with the aim of studying the distribution of the samples and their relationship with the different states of ripening. For this purpose, as in the previous sections, the preprocessed data (described in Section 2.2) were used, and the full set of samples was included in the models. The optimal number of components to build the model was 20 in all cases, after analyzing the variance for each principal component.

Figure 5 shows the PCA models for the three analytical techniques. By observing the PCA individually, it was possible to identify three different stages according to the degree of maturity of the peach: unripe peach (phase 1), midripe peach (phase 2), and ripe peach (phase 3). The temporal distribution of these phases for each of the varieties studied is shown in Figure 5D. The unripe peaches (phase 1) last until mid-July for the yellow-fleshed variety and until the first days of August for the red-fleshed variety. The second phase corresponds to the midripe peaches and lasts until the end of August for the yellow-fleshed variety and until the mid-September for the red-fleshed variety. Phase 3 peaches are those that have reached their optimum ripeness and are ready to be harvested.

**Figure 5:**
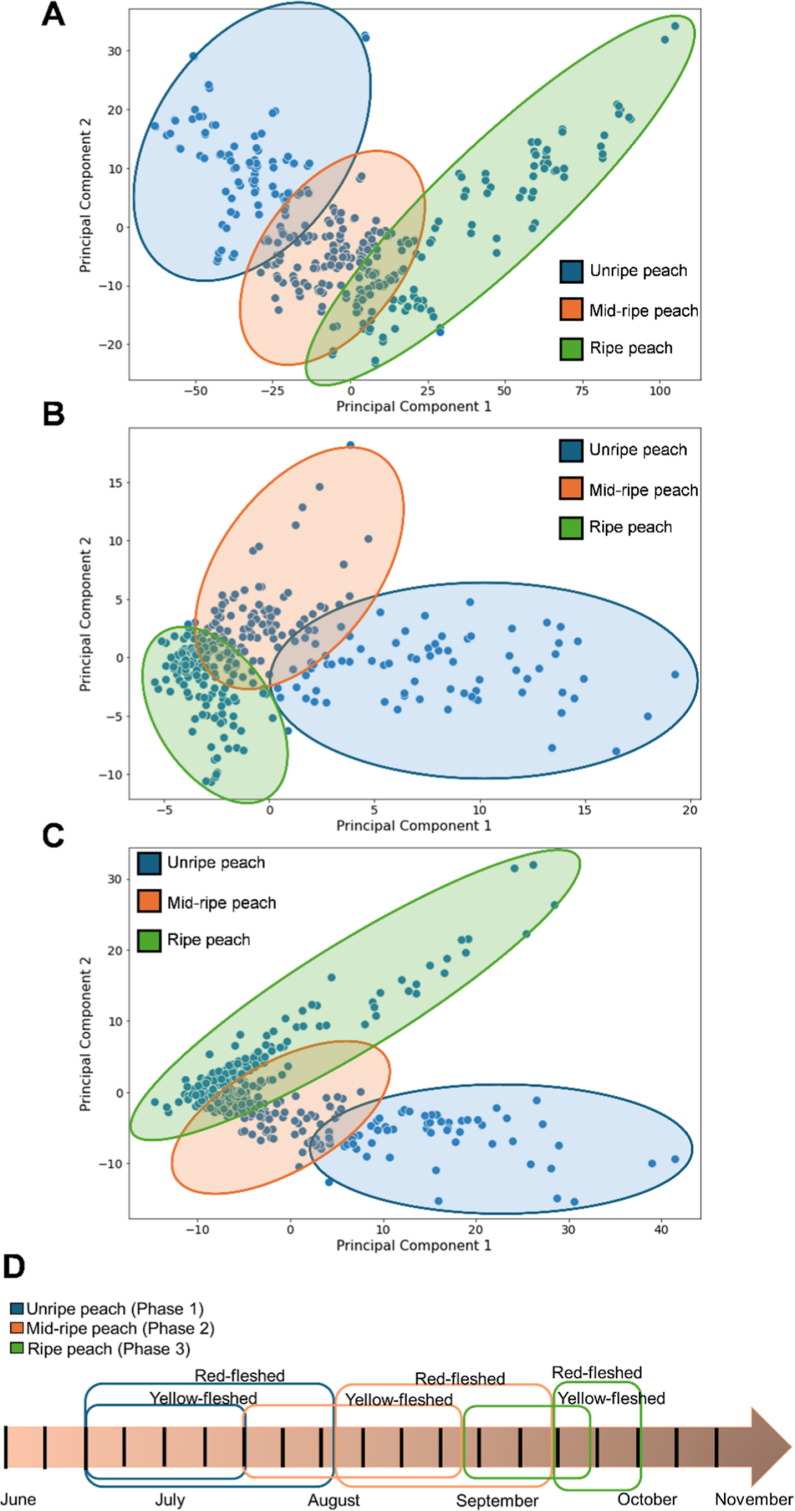
PCA score plot and classification of peaches in three stages of ripeness. Study of the evolution of spectral bands and VOCs along the ripening process applying a PCA model using the data obtained by (A) FT-NIR, (B) HS-GC–IMS, and (C) HS-GC–MS. (D) Scheme of the ripening phases identified, based on the observation of the three PCA analyses for both varieties.

Next, different PLS-DA models were constructed based on the three ripening stages established using PCA and the support of the information provided by the farmers. For the construction of these models, four possible approaches were evaluated for the three techniques used: (1) the analysis of all samples together, without distinguishing between yellow-fleshed and red-fleshed varieties (3 groups), although different ripening dates are defined for each variety; (2) the analysis of all samples together but distinguishing between varieties (6 groups); (3) the analysis of the yellow-fleshed variety (3 groups); and (4) the analysis of the red-fleshed variety (3 groups).

Table 4 shows the parameters for assessing the quality of the models built with the optimal number of components for the data extracted from the three analytical techniques with each of the four approaches studied, and in Figure 6 their PLS-DA scores.

**Table 4:** Quantitative Parameters of the Final PLS-DA Models Built to Discriminate between Maturation Stages^a^

instrument	approach	group	samples	components	A	P	Sp	Se	% *T*	CV (mean ± SD)
FT-NIR	no variety differentiation	3	344	3	90.1	90.1	94.7	90.1	91.9	83.3 ± 9.5
	two varieties	6	344	5	77.6	78.1	95.3	77.6	77.5	70.9 ± 5.3
	yellow-fleshed	3	154	2	77.9	80.1	88.5	77.9	79.1	73.9 ± 4.4
	red-fleshed	3	190	5	99.0	99.0	99.4	99.0	100	96.7 ± 2.0
HS-GC–IMS	no variety differentiation	3	344	7	95.7	95.7	97.0	95.7	99.6	94.5 ± 3.1
	two varieties	6	344	7	87.0	90.0	96.1	87.0	95.6	91.0 ± 4.6
	yellow-fleshed	3	154	7	97.4	98.3	99.0	98.1	100	98.1 ± 2.6
	red-fleshed	3	190	7	97.4	98.0	98.6	97.4	100	96.8 ± 3.1
HS-GC–MS	no variety differentiation	3	344	6	93.0	93.4	96.1	93.0	92.6	87.3 ± 3.8
	two varieties	6	344	9	94.2	94.5	98.8	94.2	95.3	90.2 ± 3.4
	yellow-fleshed	3	154	6	96.8	96.8	98.4	96.8	98.6	92.6 ± 3.2
	red-fleshed	3	190	5	96.3	96.4	98.3	96.3	97.8	94.8 ± 3.9

a A: accuracy; P: precision; Sp: specificity; Se: sensitivity; %T: training rate; CV: cross-validation; SD: standard deviation.

**Figure 6:**
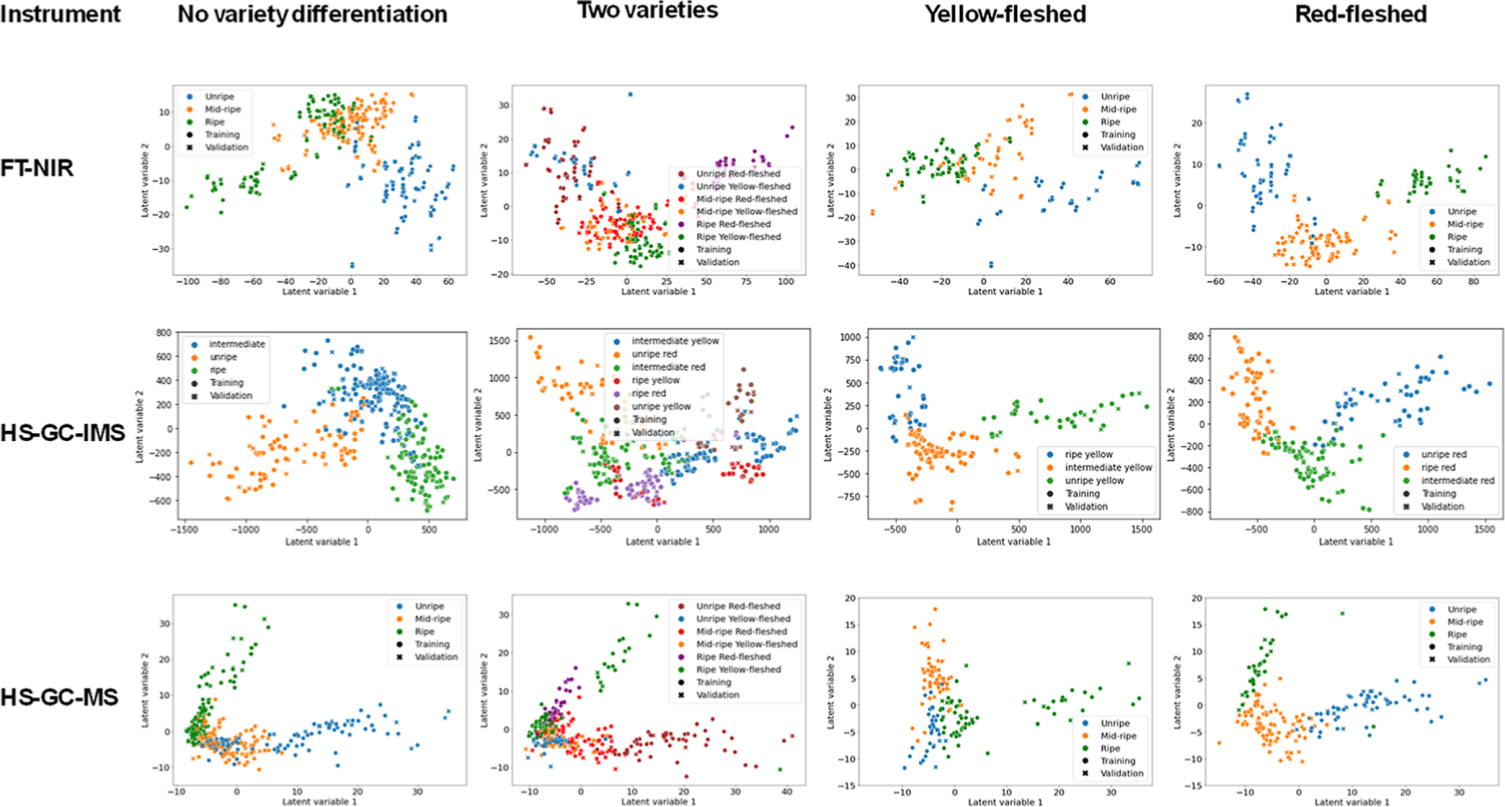
PLS-DA score plot for the differentiation of peach ripeness. Score plot obtained from the PLS-DA model built to discriminate between unripe, midripe, and ripe peaches using four different approaches: (A) FT-NIR, (B) HS-GC–IMS, and (C) HS-GC–MS.

Analyzing the results obtained from the FT-NIR analysis, the CV percentages are observed to vary between 70.9 and 96.7%. PLS-DA with the lowest percentage corresponds to the one developed with all of the samples, differentiated by variety, while the one with the highest percentage is the one developed exclusively for the red-fleshed peach samples. The same models also have the lowest and highest performance values in the training set, reaching 100% in the case of red-fleshed samples. For the parameters of accuracy, precision, specificity, and sensitivity, values between 77.6 and 99.4% are obtained in all cases. The best results, with values above 99% in all parameters, are again obtained for the PLS-DA model constructed with the red-fleshed peach samples. Some models present SDs slightly higher than 4% in the CV, which may indicate lower model stability in certain cases. Therefore, it is advisable to analyze the lower limit of this interval. In particular, the models applied to two varieties (70.9% ± 5.3%) and the yellow-fleshed variety (73.9% ± 4.4%) have lower limits below 70%, which suggests a lower generalization capacity.

The differences found in the models built for each variety separately are worth noting. These variations are explained by the two varieties’ distinct spectral profiles, as demonstrated by the PLS-DA model, which distinguishes between them with 88% CV accuracy (see Section 3.4). This discrepancy is likely due to differences in the biochemical composition of the fruits, which directly influence the signal captured by the FT-NIR technique. Therefore, it is expected that ripening prediction models adjusted to each variety will behave differently. For the red-fleshed variety, more consistent spectral variations are generated during the ripening process, which can be classified more easily by the model. In contrast, the yellow-fleshed variety may exhibit less pronounced or more heterogeneous spectral changes during ripening. This makes it challenging for the model to identify clear patterns and generate accurate predictions.

The models built from the HS-GC–IMS data showed excellent results with CVs above 91.0% in all cases. The models built independently for each variety achieved a training rate of 100% and accuracy, precision, specificity, and sensitivity values above 97%. Models including all varieties also performed well, although slightly lower (evaluation parameters ≥ 87.0%).

In the models constructed from the HS-GC–MS data, high CV percentages were observed, ranging from 87.3 to 94.8%. Moreover, the other parameters (accuracy, precision, specificity, sensitivity, and percentage of training) were above 92%. Generally, it can be concluded that all PLS-DA models showed excellent results; however, the models built for the individual peach varieties showed better performance.

Finally, the VIPs were analyzed to identify the features that contributed most to discrimination between the different maturation stages. Table S2 shows the five variables with the highest VIP values in each of the models. In the case of the models constructed from the data obtained by HS-GC–IMS, it is noteworthy that in three of the four models, pentanal (monomer and dimer) appears as one of the most relevant compounds. Furthermore, in the model generated by considering all samples without distinguishing between varieties (approach 1), (*Z*)-hexen-2-ol (monomer) and benzaldehyde (monomer and dimer) were also identified as relevant. On the other hand, in models based on HS-GC–MS data, (*Z*)-hexen-2-ol reappears as a representative compound, as in HS-GC–IMS, and terpinolene and (*Z*)-hex-3-enyl acetate are added as prominent variables (Table S2).

Therefore, the proposed PCA and PLS-DA models allow us to state that both the FT-NIR spectral data and the volatile profiles obtained by HS-GC–IMS and HS-GC–MS provide relevant information about the ripening stage of peaches. Furthermore, as in the case of differentiation between varieties, the effectiveness of these techniques and chemometric models in assessing the quality and ensuring that peaches reach an adequate degree of ripeness before harvesting has been demonstrated, especially when using the volatile profile as a characteristic, which has a greater potential to carry out this differentiation than the FT-NIR spectrum due to the greater sensitivity and specificity of the analytical techniques used for its analysis (HS-GC–IMS and HS-GC–MS). Finally, it is important to point out that the results are better for each variety separately. This makes sense because we are dealing with two completely different varieties, which, as noted in Section 3.4, are perfectly discriminated by a PLS-DA model. Therefore, in approaches 1 and 2, where all samples are considered, we work with a very large number of variables, which makes it difficult to build the PLS-DA model.

The results obtained allow us to conclude that both the quality and the specific variety of the peach can be verified by a single analysis of the volatilome or the NIR spectral fingerprint. However, better results are obtained in both cases when working with the volatile profile, since HS-GC–MS and HS-GC–IMS techniques are more powerful and specific tools. Although HS-GC–MS stands out for its high resolution and sensitivity, its high cost, the need for vacuum conditions, and the large size of the equipment limit its applicability in sectors with limited resources such as many fruit quality control companies. In contrast, the HS-GC–IMS technique offers greater simplicity, accessibility, and cost-effectiveness, as it requires less energy and resources, making it a more efficient and sustainable option.^40^ Furthermore, its ability to provide results comparable to those of HS-GC–MS, as demonstrated in this work, underscores its potential as an ideal alternative for many studies seeking to reduce resource consumption without compromising analytical quality.^41^

In summary, multiple factors must be considered to determine the best analytical tool for carrying out routine controls aimed at verifying the authenticity of labeling and predicting the optimal stage of ripening in order to prevent food fraud and minimize economic losses. These factors include economic cost, analysis time, complexity of sample preparation, and discrimination capability of the generated models. Although the FT-NIR technique allows for fast and relatively low-cost analysis, its analytical performance is significantly low. In some cases, the classification and validation percentages are close to 70%. HS-GC–MS, on the other hand, has shown very good discrimination capacity (with values above 87%), but it is limited by its high operating cost and greater instrumental complexity. Considering all these aspects, the technique that offers the most balanced approach for implementation in routine controls is HS-GC–IMS, as it combines excellent discrimination capability (with classification and validation percentages above 90% in all cases studied) with moderate analysis times, very simple sample preparation, and not very high operating costs. These features make HS-GC–IMS a particularly suitable tool for practical applications requiring a high degree of reliability, efficiency, and sustainability.

Although the results obtained were very promising, particularly regarding the volatile profiles analyzed by HS-GC–IMS and HS-GC–MS, the study has some limitations. First, the study focused on two peach varieties grown in a specific geographical region, so the applicability of the developed models to other varieties or geographical origin requires additional validation. Furthermore, while the chromatographic techniques used were successful, they call for specialized equipment, which might restrict their use in some situations. Future research should consider expanding the number of varieties and geographical origins.

## Supplementary Material



## Data Availability

Data will be made available on request.

## Notes

Optimization of the HS-GC–IMS procedure; variable importance in projection scores of the HS-GC–IMS model; VIP of PLS-DA models for peach variety discrimination; and VIP of PLS-DA models built to discriminate between maturation stages (PDF)
