# Supplementary material for: Fingerprinting of Peach During the Ripening Process Using an Analytical Platform with Spectrometric and Volatilome-Based Chromatographic Techniques
Source: J Agric Food Chem. 2025 Jun 23;73(26):16636–47. doi: 10.1021/acs.jafc.5c01524 (PMC12232294; doi:10.1021/acs.jafc.5c01524)
Supplement: Supplementary file 1 [file jf5c01524_si_001.pdf]

Fingerprinting of peach during the ripening process using an analytical platform with spectrometric and volatilome-based chromatographic techniques

Claudia Giménez-Campillo[1], Natalia Arroyo-Manzanares[1], Marta Pastor-Belda[1], Natalia Campillo[1], Lukas Bodenbender[2], Philipp Weller[2], Pilar Viñas[1,*]

[1]Department of Analytical Chemistry, Faculty of Chemistry, University of Murcia, Regional Campus of International Excellence "Campus Mare Nostrum", E-30100, Murcia, Spain

[2]Faculty of Biotechnology, Institute for Instrumental Analytics and Bioanalytics, Technische Hochschule Mannheim, 68163 Mannheim, Germany

*Corresponding author:

Prof. Pilar Viñas

Department of Analytical Chemistry

Faculty of Chemistry

University of Murcia

E-30100 Murcia

SPAIN

e-mail: pilarvi@um.es

Tel: +34-868-88-7415

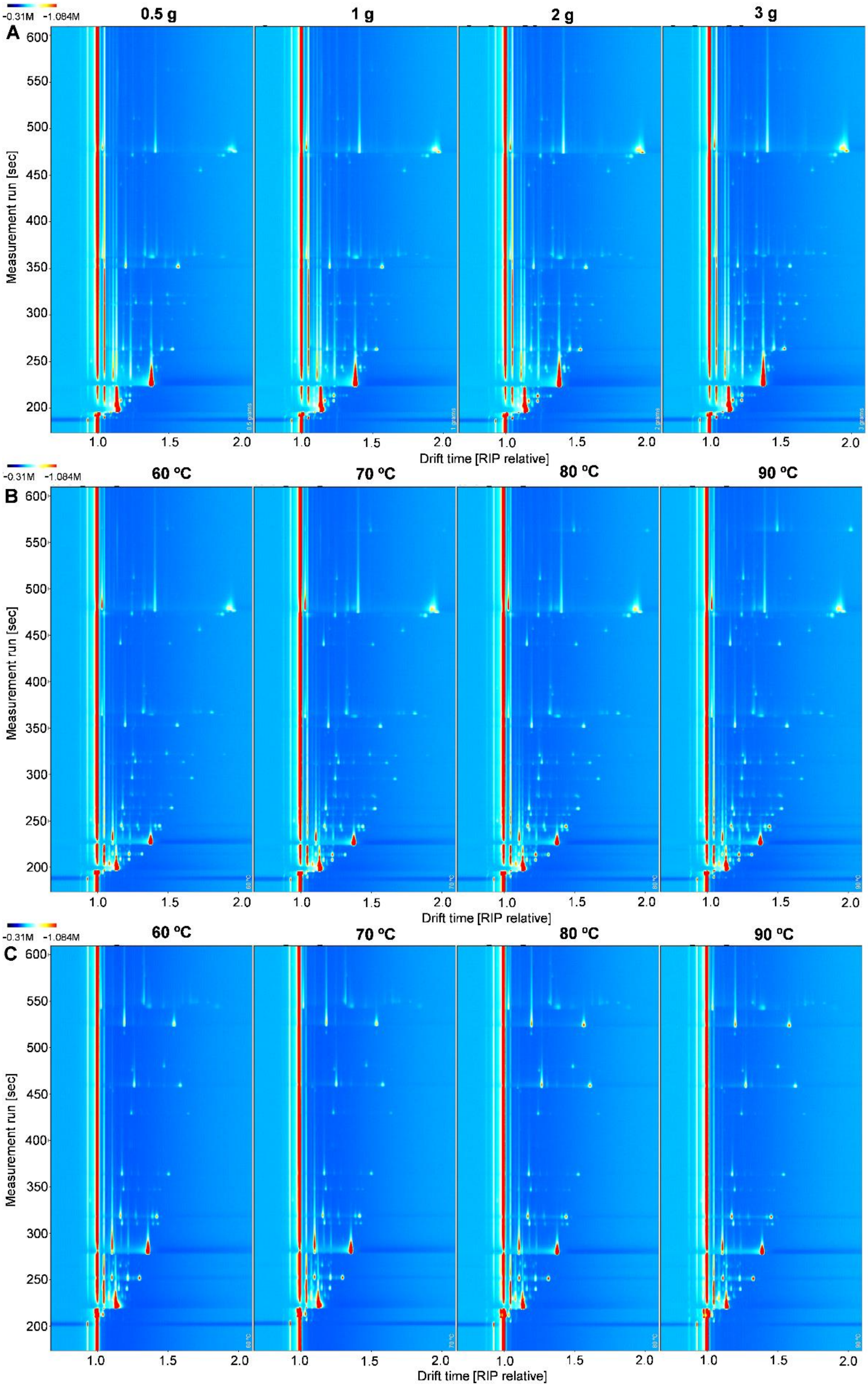

Figure S1. Optimization of the HS-GC-IMS procedure. (A) Sample amount, (B) incubation temperature and (C) temperature of the drift tube.

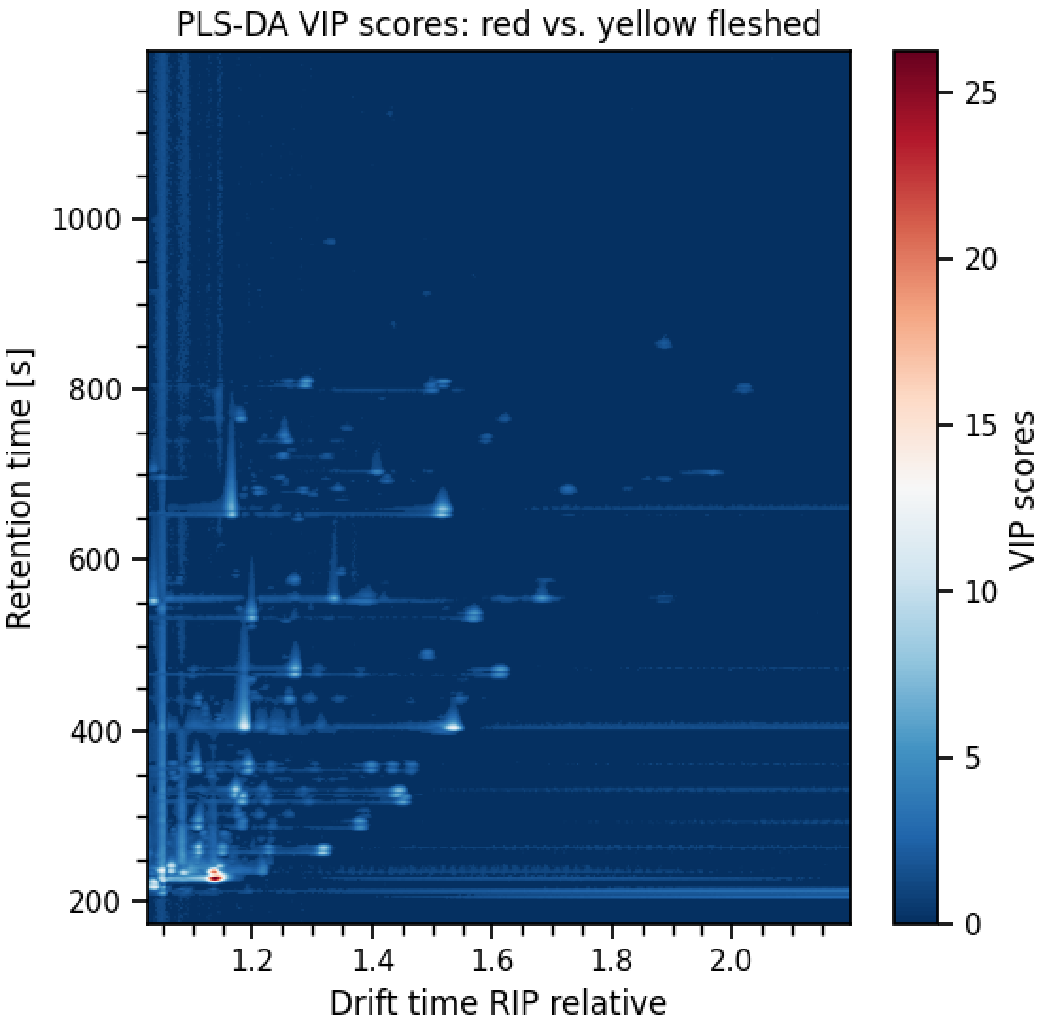

Figure S2. Variable importance in projection (VIP) scores of the HS-GC-IMS model.

Table S1. VIP of PLS-DA models for peach variety discrimination

FT-NIR	HS-GC-IMS	HS-GC-MS
3864-3876 cm^{-1}	Unknow (R_T: 227.70 s; T_D[a]: 1.134)	Unknow (R_T: 20.199 min; *m/z:* 94)
6772-6788 cm^{-1}	Unknow (R_T: 235.62 s; T_D: 1.047)	Unknow (R_T: 1.219 min; *m/z:* 29)
11044-11064 cm^{-1}	Pentanal (Dimer)	Unknow (R_T: 6.841 min; *m/z:* 207)
12004-12376 cm^{-1}	Unknow (R_T: 221.76 s; T_D: 1.036)	Unknow (R_T: 1.588 min; *m/z:* 32)
11492-11580 cm^{-1}	Pentanal (Monomer)	Unknow (R_T: 1.655 min; *m/z:* 26)

[a]T_D: relative drift time of the model.

Table S2. VIP of PLS-DA models built to discriminate between maturation stages

	No variety differentiation	Two varieties	Yellow-fleshed	Red-fleshed
**FT-NIR**	11320-11348 cm^{-1}	7252-7356 cm^{-1}	11312-11324 cm^{-1}	3676-3860 cm^{-1}
	12336-12348 cm^{-1}	8200-8216 cm^{-1}	12232-12244 cm^{-1}	10932-1136 cm^{-1}
	3752-3844 cm^{-1}	6032-6092 cm^{-1}	11192-11196 cm^{-1}	6872-6948 cm^{-1}
	6008-6016 cm^{-1}	3756-3772 cm^{-1}	12000-12080 cm^{-1}	11636-11652 cm^{-1}
	11648-11652 cm^{-1}	11496-11500 cm^{-1}	11616-11756 cm^{-1}	11824-11964 cm^{-1}
**HS-GC-IMS**	Pentanal (Dimer)	Unknow (R_T: 227.70 s; T_D[a]: 1.137)	Unknow (R_T: 235.62 s; T_D: 1.134)	Unknow (R_T: 225.72 s; T_D: 1.136)
	Pentanal (Monomer)	Pentanal (Dimer)	Unknow (R_T: 225.72 s; T_D: 1.136)	Pentanal (Dimer)
	(Z)-Hexen-2-ol (Monomer)	Unknow (R_T: 235.62 s; T_D: 1.049)	Unknow (R_T: 257.40 s; T_D: 1.139)	Pentanal (Monomer)
	Benzaldehyde (Dimer)	Pentanal (Monomer)	Unknow (R_T: 324.72 s; T_D: 1.443)	Unknow (R_T: 263.34 s; T_D: 1.319)
	Benzaldehyde (Monomer)	Unknow (R_T: 237.60 s; T_D: 1.080)	Unknow (R_T: 326.70 s; T_D: 1.173)	Unknow (R_T: 223.74 s; T_D: 1.033)
**HS-GC-MS**	(Z)-Hex-3-enyl acetate	Unknow (R_T: 20.199 min; *m/z:* 94)	Unknow (R_T: 8.474 min; *m/z:* 77)	Unknow (R_T: 4.765 min; *m/z:* 92)
	Unknow (R_T: 4.765 min; *m/z:* 92)	Unknow (R_T: 4.095 min; *m/z:* 58)	Unknow (R_T: 8.577 min; *m/z:* 57)	Unknow (R_T: 2.269 min; *m/z:* 83)
	Terpinolene	Unknow (R_T: 2.269 min; *m/z:* 83)	Unknow (R_T: 14.787 min; *m/z:* 91)	Unknow (R_T: 4.441 min; *m/z:* 43)
	Unknow (R_T: 1.775 min; *m/z:* 67)	Unknow (R_T: 4.068 min; *m/z:* 57)	Unknow (R_T: 7.912 min; *m/z:* 69)	Unknow (R_T: 4.095 min; *m/z:* 58)
	Unknow (R_T: 14.013 min; *m/z:*105)	Unknow (R_T: 4.047 min; *m/z:* 43)	(Z)-Hexen-2-ol	Unknow (R_T: 4.068 min; *m/z:* 57)

[a]T_D: relative drift time of the model.
